# Nitric oxide: a brief overview of chemical and physical properties relevant to therapeutic applications

**DOI:** 10.4155/fso.15.59

**Published:** 2015-08-01

**Authors:** Jack R Lancaster

**Affiliations:** 1 Departments of Pharmacology & Chemical Biology, Medicine, & Surgery, School of Medicine, University of Pittsburgh, 4200 Fifth Ave, Pittsburgh, PA 15260, USA

**Keywords:** diffusion, oxidation-reduction, radical, transition metals

## Abstract

Nitric oxide (nitrogen monoxide, •NO) has been intensively studied by chemists and physicists for over 200 years and thus there is an extensive database of information that determines its biological actions. This is a very brief overview of the chemical and physical properties of •NO that are most relevant to Biology in general and to the development of •NO releasing materials in particular.

**Figure 1.**
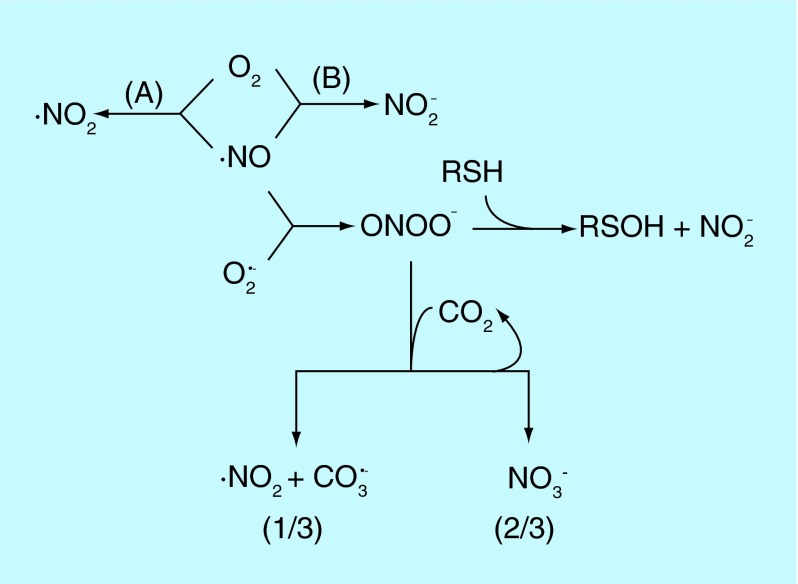
**The preponderant covalent reactions of •NO and progeny under biological conditions.** Reactions not balanced chemically. **(A)** should be very minor except at very high (•NO), but could be more important in specific microenvironments; product may not be ‘authentic’ •NO_2_. **(B)** Mechanism(s) unknown; probably second most preponderant biological •NO reaction (behind O_2_Hb reaction).

**Figure 2.**
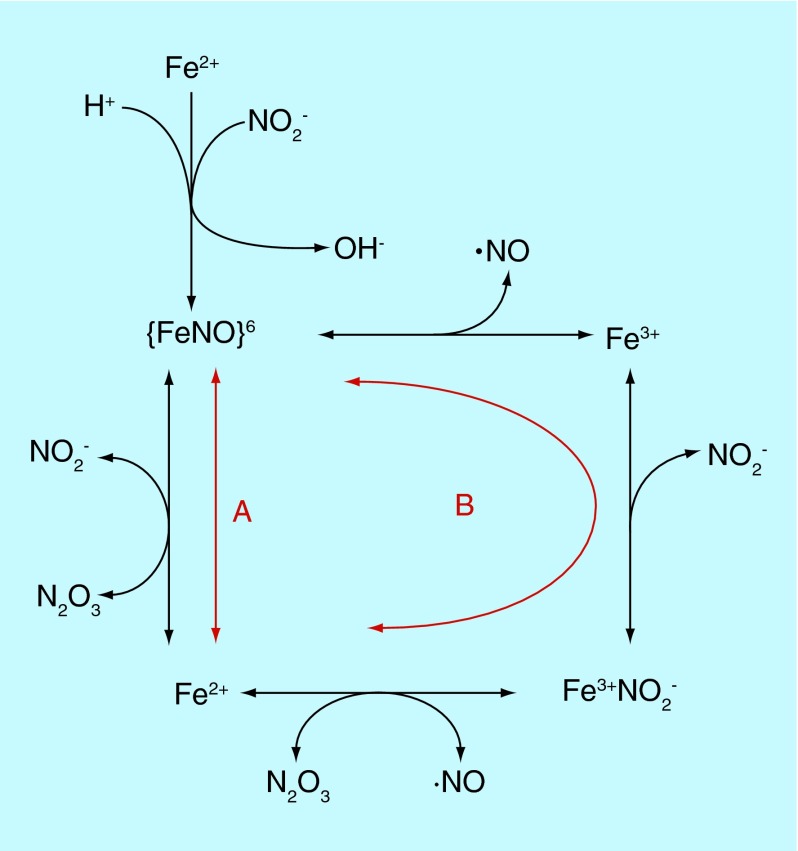
**Possible mechanisms for formation of N_2_O_3_ by reaction of nitrite with deoxyhemoglobin.** Two mechanisms are denoted by ‘A’ and ‘B,’ although both may occur at appreciable rates.

In 1987 the startling discovery was made that the small reactive radical molecule nitric oxide (•NO; nitrogen monoxide) is specifically produced and functions as a major messenger in the mammalian cardiovascular system, for which the 1998 Nobel Prize in Physiology or Medicine was awarded to Robert Furchgott, Louis Ignarro and Ferid Murad [1]. This discovery represented a classical paradigm shift in chemical biology, revealing the profound concept that Nature utilizes to its advantage the properties of molecules, even if they can also be injurious or toxic. Prior to this realization, •NO was known to biologists almost exclusively as only a toxic pollutant and we now know it is truly a versatile player in virtually every aspect of physiology and pathophysiology.

The proposition that the fundamental principles of biology must ultimately be determined by the interactions of atoms and molecules with each other is no better illustrated than examining the mechanistic foundations of the biological actions of •NO. Because •NO has been studied since the dawn of chemistry and is one of the ten smallest stable molecules in Nature [2], understanding of its basic chemistry is relatively well developed. The three properties that most determine this chemistry are that •NO is small, uncharged and (unlike virtually all other molecules) contains an unpaired electron. This is due to the fact that it has an odd total number of electrons (15). The property of possessing an unpaired electron is called paramagnetism and it is this property that most determines its chemical reactivity. However, the small uncharged nature of •NO is especially important in determining mechanisms for its selective delivery, the topic of this monograph.

This brief overview is organized into two general topics, the chemical and the physical properties of •NO relevant to its biological actions.

## Chemical properties of •NO: a lone electron in search of a stable home

Unpaired electrons in σ or π molecular orbitals are inherently unstable. The two methods for the stabilization of this electron are pair with another unpaired electron and take up partial residence in a *d* orbital of a transition metal ion. These two mechanisms dominate the chemistry of •NO, as described below.

### Pairing of the unpaired electron

One of the most important principles in chemistry is that electrons exist in atoms and molecules almost exclusively in pairs. This means of course that the unpaired electron in •NO will require another unpaired electron, which explains the rich chemistry of the reaction of •NO with other radical species. This also explains why •NO is relatively unreactive to virtually all molecules in biology. These principles provide clarity to statements that •NO is ‘reactive’ or ‘unreactive.’ In reality, •NO does not react with almost all molecules (in that sense being ‘unreactive’) but reaction with other radical molecules is very rapid ('reactive') because it involves the very favorable pairing of two unpaired electrons. Figure 1 diagrams the quantitatively most important biological reactions of •NO with nonmetals and its subsequent reactive nitrogen species (RNS) [3].

Among the biological molecules with unpaired electrons, molecular oxygen (O_2_) is pre-eminent. Although O_2_ has an even total number of electrons, in the most common form (the ground state) two of the electrons are unpaired (occupy separate molecular orbitals) and so the reaction of •NO with O_2_ (Figure 1A) is without doubt the single most studied reaction of •NO. However, in spite of this attention (extending back to John Dalton, the ‘Father of Chemistry,’ who used it to perform the first determination of the O_2_ content of the atmosphere [4]) there are many aspects of this complex reaction that are not understood. The immediate product is generally (although not universally [5]) believed to be another small radical molecule, nitrogen dioxide (^•^NO_2_). Although •NO is not an oxidant, ^•^NO_2_ definitely is and it reacts rapidly with many molecules ‘rich’ in electrons (nucleophiles) to extract one electron, generating another radical and forming nitrite anion (NO_2_^-^) from the •NO_2_. This •NO/O_2_ reaction exhibits unusual kinetics in that the rate is second order with •NO concentration. This means the rate decreases exponentially with decreasing •NO concentration, and at the nanomolar concentrations of •NO biologically (except perhaps under some inflammatory conditions) this reaction is extremely slow and likely to be of very limited importance [4]. There however may be specific cellular microenvironments (specifically the hydrophobic phases of membranes and lipid particles) where the reaction is accelerated due to increased solubilities of both •NO and O_2_ in these phases [6]. A much more important route quantitatively to biological formation of nitrite from •NO is an ill-defined process (Figure 1B), whereby cells consume •NO and O_2_ [7]. This reaction may well be the second most important biological reaction of •NO quantitatively (second to reaction with oxyhemoglobin, *vide infra*). The mechanism(s) and intermediates are unknown.

•NO also reacts with the one electron reduced form of oxygen, superoxide (O_2_^•^^-^), and unlike the O_2_ reaction is extremely rapid and kinetically first order with [O_2_^•^^-^]. In this case, the immediate product, peroxynitrite (ONOO^-^), is not a radical but it is a moderately strong two-electron oxidant. Under biological conditions the major reaction of ONOO^-^ is with CO_2_. This reaction produces a very transient species (nitrosoperoxocarbonate, ONOOCO_2_^-^), 2/3 of which forms the relatively unreactive nitrate anion (NO_3_^-^) and also regenerates CO_2_ and 1/3 rapidly homolyzes (breaks the peroxo O-O bond with each electron going to separate products) to form two radicals, ^•^NO_2_ and carbonate radical anion (CO_3_^•^^-^). Thus, •NO essentially converts 1/3 of a relatively mild oxidant (O_2_^•^^-^) into two highly oxidizing radicals. Under biological conditions another reaction of peroxynitrite that occurs, although second quantitatively to CO_2_, is with cellular thiols (RSH) to result in a two-electron oxidation to form the moderately reactive sulfenic acid (RSOH). Finally, in certain microenvironments protonated peroxynitrite (peroxynitrous acid, ONOOH; pKa = 6.8) homolyzes to produce ^•^NO_2_ and the very highly oxidizing hydroxyl radical (^•^OH).

The overall ‘take home lesson’ is that the initial reactions of •NO with nonmetals under biological conditions results in two major effects, oxidation of cellular nucleophiles and formation of the relatively stable nitrite and nitrate anions. The two major nucleophiles that are best studied are cysteine thiol (proteins and also the small peptide glutathione, GSH) and the phenolic ring of tyrosine. One-electron oxidation by the radicals (^•^NO_2_, CO_3_^•^^-^ and, to a lesser extent, ^•^OH) generates the thiyl and tyrosyl radicals. These can be considered ‘first generation’ radical products because they, like •NO, become stabilized primarily by reaction with other molecules with unpaired electrons. Thiyl radical reaction with •NO will result in nitrosothiol, a covalent compound containing the nitroso functional group (R-S-N = O). Tyrosyl radical reaction with •NO_2_ will result in nitrotyrosine, a covalent compound with the nitro functional group (R-NO_2_). These are by far the two best studied nitrogen oxide adducts formed from •NO biologically. Another commonly cited mechanism for RSNO formation involves the reaction of •NO_2_ with •NO to yield nitrous anhydride (N_2_O_3_) which reacts with thiol (and also H_2_O) by transfer of the nitroso group (transnitrosation). Although this reaction dominates the chemical reactivity of •NO in pure solution under aerobic conditions, it is almost certainly unimportant biologically (except perhaps in specific microenvironments [6]) because it is much more likely that the •NO_2_ will react by one-electron oxidation with nucleophile (*e.g.*, GSH which is present in cells at up to 10 mM concentrations) than to react with the nanomolar concentrations of •NO [8]).

### Partial residence in transition metal *d* orbitals

Transition metals are so named because in the periodic table they represent ‘transition’ elements between the *s* and *p* orbitals, with electrons (both paired and unpaired) occupying *d* orbitals. These orbitals can accommodate up to 10 electrons and in general can be fairly closely spaced in energy, depending on the arrangement of ligands around the metal. These properties mean that transition metals are excellent ‘homes’ to unpaired electrons. However, although complete electron transfer from a radical can occur, it is more common that the unpaired electron(s) is shared by the radical molecule and the transition metal. Sharing of electrons by atoms and molecules is of course called a ‘bond’ so in particular •NO and O_2_ both form bonds to transition metals. The bond that is formed is distinct from an organic covalent bond and is called a coordinate bond.

The best studied transition metal which bonds •NO is iron, which bonds in either the ferrous (Fe^2+^) or ferric (Fe^3+^) state. The dissociation of •NO from ferroheme proteins is in general much slower than for ferriheme proteins, so •NO is bound much more tightly to ferroheme. Since the coordinate bond is so different from the covalent bond there are different terminologies for the nitrogen oxide, nitroso for the functional group in covalent compounds and nitrosyl for the ligand bound to transition metal.

### Reactions involving both pairing of unpaired electrons & transition metals

Because the iron in many hemoproteins is redox active it can participate in one-electron oxidation-reductions of •NO. In fact, undoubtedly the most important reaction of •NO biologically (quantitatively) is the extremely rapid reaction with oxyferrohemoglobin to form nitrate and ferriheme:Equation 1
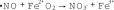



This electron transfer can also occur in reverse, with electron flow to the ferriheme to form ferroheme; this is the basis for the formation of •NO from nitrite, which is in the same formal oxidation state as oxidized •NO, nitrosonium(NO^+^; nitrosonium will never exist as a free ion under biological conditions because of extremely rapid reaction with H_2_O):Equation 2
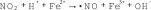



It is important to note that any mechanism that results in formation of •NO from nitrite will always involve consumption of a proton and so commonly will be more rapid as the pH is lowered. This is true also when transition metal is not involved, for example, the well known reactions that occur upon acidification of nitrite involving nitrous anhydride (N_2_O_3_) [9]:Equation 3
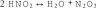

Equation 4
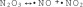



The net effect of this reaction sequence is electron transfer from one nitrite to another, forming •NO_2_ (the one-electron oxidized product of nitrite) and •NO (the one-electron reduced product of nitrite).

There also are proposals involving a reaction sequence associated with both heme iron oxidation-reduction and pairing-unpairing of electrons in nitrogen oxides [10,11]. The biological conundrum these schemes are meant to address is how the vasodilatory activity of nitrite, apparently attributable to reduction to •NO by deoxyhemoglobin, is preserved when the •NO is produced within the red cell, an environment containing massive concentrations of an extremely rapid scavenger (oxyhemoglobin, even in relatively hypoxic areas). The essential feature of these schemes is the notion that the •NO is ‘shielded’ from scavenging in the form of N_2_O_3_, which is produced by hemoglobin-catalyzed anhydration (loss of water) from two molecules of nitrite. The N_2_O_3_ could escape the red cell and homolyze, forming •NO (and •NO_2_) and thus inducing dilation. The overall nitrogen oxide chemistry (reactants and products) is identical to formation of •NO from acid nitrite (Equation 3). The two mechanisms that have been proposed for deoxyhemoglobin catalysis are illustrated in Figure 2, denoted by ‘A’ [10] and ‘B’ [11].

Consideration of this mechanism illustrates the utility of the Enemark-Feltham notation for designation of iron-nitrosyl complexes [12], which is {Fe(NO)_x_}^y^ where ‘x’ denotes the number of nitrosyls coordinated to the iron and ‘y’ is the number of electrons in the *d* orbitals of the iron plus the number of unpaired (π*) electrons from the •NO(s). In this case, the iron-nitrosyl is formed from nitrite binding to a ferrous iron, accompanied by uptake of a proton and loss of hydroxide (anhydration). Since the nitrogen oxide in this case is formally one-electron oxidized •NO (protonated nitrite can be considered formally as nitrosonium hydroxide, [NO^+^][OH^-^]), this complex is thus designated {FeNO}^6^ because no unpaired electron is contributed by the nitrogen oxide and the iron contains six d electrons. The identical complex can also be formed from binding of •NO (one unpaired electron) to ferric iron (five unpaired electrons). According to molecular orbital theory the chemical identity of the complex is the same ({FeNO}^6^) no matter which mechanism is responsible for formation; in essence, it makes no difference where the electrons were located in the reactants because in the complex they are all shared by both the iron and the nitrogen oxide (the very definition of ‘molecular orbitals’).

Much of the basis of these proposals is derived from work by Ford [13] on the mechanism of a phenomenon first reported in 1937 [14] that addition of •NO to ferriheme proteins results in the formation of the ferroheme complex and is termed ‘reductive nitrosylation'. The mechanism involves the oxidation of •NO to a nitrosating species which can accomplish the nitrosation of nitrite to form N_2_O_3_. There is at present no consensus as to which mechanism ‘A’ or ‘B’ is responsible for the formation of N_2_O_3_ from nitrite and it is quite possible that both occur at appreciable rates.

## Physical properties of •NO: a lone molecule in search of a sink

The metaphor of a molecule involved in a ‘search’ for a target is useful as a didactic teaching tool, but it suffers from the serious caveat that it is fallaciously anthropomorphic. That is, molecules are of course nonsentient and in solution neither travel in linear paths nor purposely pursue a specific direction (the common usage (including by the author) of straightlines in schematic diagrams unfortunately tends to exacerbate this misconception). The movement of •NO is by random diffusion, meaning the probability of displacement in any direction is equal to the probability in any other direction.

Even though an individual molecule is equally likely to move in any direction, collections of molecules can certainly move in a certain direction. This will occur if there are concentration gradients and it is a result of the fact that the probability of movement between two compartments with different concentrations is greater for movement into the location with lower concentration and the result is that net movement will occur down a gradient. Thus, a collection of •NO molecules will always move away from the point at which it is made and in the direction of a sink. Since •NO is a very small and uncharged molecule it has a high diffusion constant and will travel great distances in a very short time [15]. However, the presence of a rapid sink will greatly confine its diffusion field if located in proximity to the source.

**Executive summary**

The chemistry of •NO is determined by the fact that it is a radical (contains an unpaired electron).
•NO reacts at appreciable rates with only two molecular targets, molecules that possess unpaired electrons and transition metals.
The chemistry of •NO is dominated by oxidation-reduction reactions, which can produce other nitrogen oxide species with much higher reactivity that •NO itself.
•NO binds avidly to transition metals and can undergo further oxidation-reduction and bond formation/breakage.
•NO is highly diffusible in the biological milieu.
